# Performance of an Electronic Sentinel Surveillance System for Bacterial Gastroenteritis: Mixed Methods Evaluation Study

**DOI:** 10.2196/102373

**Published:** 2026-08-26

**Authors:** Inmyung Song, Yerin Heo, Minsol Jo, Jung-yeon Park, Sukhyun Ryu, Dong-Sook Kim

**Affiliations:** 1 College of Nursing and Health, Kongju National University, Gongjudaehakro 56, Gongju, Chungcheongnam-do, 32588, Republic of Korea, 82 41-850-0325, 82 41-850-0320; 2 Department of Preventive Medicine, College of Medicine, The Catholic University of Korea, Seoul, Republic of Korea

**Keywords:** bacterial gastroenteritis, sentinel surveillance, public health surveillance, electronic reporting, claim data, data linkage, evaluation, South Korea, notifiable diseases, foodborne illness

## Abstract

**Background:**

Bacterial gastroenteritis remains a substantial public health burden. South Korea has operated an electronic sentinel surveillance system (SSS) since 2000; in 2023, a total of 211 sentinel hospitals conducted surveillance for 11 notifiable types of bacterial gastroenteritis. The system has not undergone a comprehensive evaluation.

**Objective:**

This study evaluated the SSS’s operational performance and reporting completeness, assessed the population-based geographic allocation of sentinel sites, and explored concordance between SSS reports and National Health Insurance (NHI) claim records.

**Methods:**

We conducted a mixed methods evaluation using a 2024 survey of staff at the 211 sentinel hospitals, an interview with the Korea Disease Control and Prevention Agency (KDCA) operator, KDCA operational reporting records, nationwide NHI claim records, and regional population statistics. The primary evaluation year was 2023; 2021 was a nonconsecutive descriptive comparator because these were the 2 complete annual extracts available in the analytic dataset. We calculated delayed reporting, submission completeness, and on-time completeness. Exploratory Pearson correlations compared paired weekly all-pathogen totals (52 weeks per year), annual surveillance target, and diagnosis code category counts within each age group (11 paired categories), and actual vs population-proportional sentinel allocations across 17 regions.

**Results:**

Of 211 sentinel hospitals, 180 (85.3%) responded. In total, 90% (162/180) rated reporting as simple or adequate; responses on goal awareness and overall satisfaction were predominantly neutral. The delayed reporting proportion was 2.4% (255/10,652) in 2021 and 1.1% (122/10,972) in 2023; submission completeness was 99.9% (10,652/10,660) and 100% (10,972/10,972), respectively; and on-time completeness was 97.5% (10,397/10,660) and 98.9% (10,850/10,972), respectively. The exploratory weekly SSS-NHI correlation was 0.899 in 2021 and 0.937 in 2023. Age-stratified annual cross-category correlations ranged from 0.665 to 0.969 in 2021 and from 0.939 to 0.983 in 2023. Correlations between actual and population-proportional sentinel allocations were 0.946 in 2021 and 0.951 in 2023.

**Conclusions:**

The SSS demonstrated favorable operational performance, with low reporting delay and near-complete submission. The claim comparison showed strong ecological comovement but cannot establish sensitivity or diagnostic equivalence because the data sources use different case definitions and counting units. Priorities are clearer communication of operational goals, standardized operator training, and future validation using patient- or episode-level data linked to laboratory results.

## Introduction

Gastroenteritis is caused by a wide range of pathogens, including bacteria, viruses, and parasites, that infect the gastrointestinal tract [1]. The causal pathogen is often not identified or reported [2], yet bacterial enteric pathogens are responsible for a large share of the burden, accounting for an estimated 20% to 40% of diarrhea cases worldwide [3,4]. The downstream consequences are striking: enteric infectious diseases caused an estimated 1.27 million deaths worldwide in 2023, with diarrheal disease accounting for most of these deaths (1.11 million) [5], whereas in high-income settings, acute gastroenteritis drives substantial outpatient and inpatient costs [6,7].

South Korea is no exception to this burden. Health insurance claims for 18 foodborne pathogens recorded more than 300,000 cases annually between 2008 and 2012 [8]. Among Korean children between 2004 and 2019, bacterial pathogens were responsible for 3% to 20% of acute gastroenteritis cases, with *Escherichia coli* and *Salmonella* spp. predominating [9]. By 2023, bacterial gastroenteritis accounted for half of all gastroenteritis cases reported to the Korea Disease Control and Prevention Agency (KDCA); *Salmonella* spp., *Campylobacter* spp., and enteropathogenic *E. coli* were the most frequently notified pathogens [10]. The case for close monitoring is likely to intensify: South Korea’s summers are growing warmer and longer, which favors bacterial transmission [11], and rising international travel has made surveillance of previously unreported pathogen genotypes increasingly important [9].

South Korea introduced a sentinel surveillance and electronic reporting system in 2000 [12] targeting infectious diseases with high incidence but low severity [13]. By 2023, a total of 11 types of bacterial gastroenteritis were classified as group 4 notifiable diseases and had to be reported to the health authority within 7 days of diagnosis through the sentinel surveillance system (SSS) [13]. Today, 211 sentinel hospitals submit weekly case reports to the KDCA through this nationwide network. Surveillance systems for gastrointestinal diseases vary widely worldwide in design and data source, ranging from laboratory-based and sentinel-clinician networks to syndromic and administrative claim–based approaches; a recent systematic review found such systems to be validated for routine use in at least 10 countries [14]. A previous evaluation looked at the SSS for hand, foot, and mouth disease [15], but to our knowledge, no comprehensive evaluation has yet examined the SSS specifically for bacterial gastroenteritis. Closing this gap matters: routine surveillance for this disease group needs to be assessed on its own terms, and concrete strategies for improvement need to be identified.

To address this gap, we conducted a mixed methods evaluation of South Korea’s electronic SSS for bacterial gastroenteritis with 2023 as the primary evaluation year and 2021 as a nonconsecutive descriptive comparator. The objectives were to (1) assess simplicity, acceptability, timeliness, submission completeness, and on-time completeness; (2) examine whether sentinel site allocation across 17 regions was proportional to population size; and (3) explore whether weekly all-pathogen totals and annual age-stratified surveillance target or diagnosis code category patterns in SSS reports were concordant with National Health Insurance (NHI) claim records.

## Methods

### Study Design

This mixed methods surveillance system evaluation combined a cross-sectional survey and structured operator interview with retrospective analysis of operational reporting records, NHI claim records, and regional population statistics. The primary evaluation year was 2023, with 2021 used only as a nonconsecutive descriptive comparator. The operational and external comparison components were analyzed separately so that claim data were not treated as a reference standard for laboratory-confirmed SSS cases.

### Data Sources

#### Overview

From July 29, 2024, to August 30, 2024, we emailed a semistructured questionnaire to the staff member responsible for case notification at each of the 211 sentinel hospitals. Items covered the perceived simplicity of reporting, awareness of SSS operational goals, overall satisfaction, and mean weekly reporting time. From these 211 hospitals, we received 180 (85.3%) completed questionnaires.

We conducted a structured interview with the KDCA operator responsible for routine SSS operation. Topics included reporting compliance, ease of data collection and dissemination, recurrent data quality problems, operator workload, and whether formal training or standardized operating materials were provided. Separately, KDCA operational extracts supplied the number of designated sentinel sites, facilities with no laboratory-confirmed case notifications for more than 6 months (an indicator distinct from missing scheduled weekly submissions), and weekly on-time and delayed submission counts for 2021 and 2023.

Nationwide NHI claim records were obtained from the Health Insurance Review and Assessment Service for January 2021 to December 2021 and January 2023 to December 2023. Variables included *International Classification of Diseases, 10th Revision* (*ICD-10*), diagnosis code and position; outpatient or inpatient status; age group; treating institution region; service date; and billed laboratory test procedures. Official resident registration population counts for the 17 administrative regions in the corresponding years were obtained from the Korean Statistical Information Service and were used only to calculate population-proportional expected numbers of sentinel sites [16].

Table 1 lists the 11 types of gastroenteritis that are notifiable through the SSS, with their causative pathogens and diagnostic criteria [17].

**Table 1.** Laboratory-confirmed types of bacterial gastroenteritis included in South Korea’s electronic sentinel surveillance system (SSS) and national SSS case counts in 2023.

Diseases	Causative pathogen	Diagnostic criteria	KDCA^a^-reported cases in 2023, n
Salmonellosis	Nontyphoidal *Salmonella* spp.	Isolation and identification of nontyphoidal *Salmonella* spp. from specimens (stool and rectal swabs)	3540
*Vibrio parahaemolyticus* gastroenteritis	*V parahaemolyticus*	Isolation and identification of *V parahaemolyticus* from specimens (stool and rectal swabs)	101
ETEC^b^	ETEC	Isolation and identification of *Escherichia coli* possessing heat-labile toxin genes (*LT*) or heat-stable toxin genes (*ST*) from specimens (stool and rectal swabs)	481
EIEC^c^	EIEC	Isolation and identification of *E. coli* possessing the invasive factor gene (*ipaH*) from specimens (stool and rectal swabs)	55
EPEC^d^	EPEC	Isolation and identification of *E. coli* possessing adherence factor genes (*eaeA* and *bfpA*) from specimens (stool and rectal swabs)	1963
Campylobacteriosis	*Campylobacter jejuni* and *Campylobacter coli*	Isolation and identification of *Campylobacter* spp. from specimens (stool, rectal swabs, and vomitus)	3167
*Clostridium perfringens* enteritis	*C. perfringens*	Detection of *C. perfringens* at levels of ≥10^6^ CFU^e^ per gram in specimens (stool and vomitus) or isolation and identification of *C. perfringens* possessing enterotoxin-specific genes (*cpa* and *cpe*) from specimens (stool, rectal swabs, and vomitus)	423
*Staphylococcus aureus* intoxication	*S. aureus*	Isolation and identification of *S. aureus* possessing enterotoxin genes from specimens (stool, rectal swabs, and vomitus)	160
*Bacillus cereus* gastroenteritis	*B. cereus*	Isolation and identification of *B. cereus* possessing toxin genes (*hblC*, *nheA*, *entFM*, *cytK2*, *becT*, and *CER*) from specimens (stool, rectal swabs, and vomitus)	42
Yersiniosis	*Yersinia enterocolitica*	Isolation and identification of *Y. enterocolitica* from specimens (stool, rectal swabs, and vomitus)	169
Listeriosis	*Listeria monocytogenes*	Isolation and identification of *L. monocytogenes* from specimens (stool and rectal swabs)	10

a KDCA: Korea Disease Control and Prevention Agency.

b ETEC: enterotoxigenic *E. coli*.

c EIEC: enteroinvasive *E. coli*.

d EPEC: enteropathogenic *E. coli*.

e CFU: colony-forming unit.

#### Case Identification in NHI Claim Data

To identify NHI claim records corresponding to the 11 SSS targets, we used *ICD-10* codes A02, A04, A05, and A32 and their pathogen-specific subcodes where available. These diagnosis code categories correspond to salmonellosis, other bacterial intestinal diseases, bacterial foodborne intoxications, and listeriosis. They do not establish microbiological identification of the causative pathogen. Some parent codes encompass more than one organism; therefore, category-level analyses compared SSS surveillance targets with corresponding diagnosis code categories and should not be interpreted as one-to-one pathogen matching.

Counts were extracted at the claim record level rather than at the unique patient or episode level; repeated outpatient or inpatient claims for the same person could therefore be included. To increase coding specificity, we retained primary or secondary diagnosis records accompanied by a billed laboratory test procedure and excluded follow-up records without an active diagnosis code. A billed test indicates only that testing was ordered or claimed; the result is not available in NHI data. Consequently, the algorithm may include empirically treated or miscoded records and is not equivalent to the laboratory-confirmed SSS case definition. We did not use these data to calculate incidence, case count ratios, capture fractions, or diagnostic accuracy. Throughout the manuscript, “claim records” denotes NHI observations, and “cases” denotes SSS reports.

### Study Period

The primary evaluation year was 2023 because it was the most recent complete year in the project dataset and was closest to the 2024 survey and operator interview. The analytic dataset also contained a complete 2021 extract, which was retained as a nonconsecutive historical comparator; 2022 was not included in the extract available to this study. We did not treat 2021 as an unaffected prepandemic baseline. Comparisons between 2021 and 2023 are descriptive snapshots, and no continuous temporal trend or causal effect of the COVID-19 pandemic was inferred.

### Evaluation Framework

#### Overview

Our evaluation used the KDCA guidelines [18], which adapt the US Centers for Disease Control and Prevention framework for evaluating public health surveillance systems [19]. The framework had previously been applied to another South Korean SSS [15]. We assessed operational performance (simplicity, acceptability, and timeliness), reporting completeness (submission completeness and on-time completeness), and geographic allocation. Because sensitivity requires a valid denominator or reference standard and NHI claims do not provide one, sensitivity was not estimated. Weekly and age-stratified claim comparisons were analyzed as a separate exploratory external concordance component. Table 2 shows the operational definitions and data sources.

**Table 2.** Operational definitions and data sources for the mixed methods evaluation of South Korea’s electronic bacterial gastroenteritis sentinel surveillance system (SSS) using 2021 and 2023 operational and claim data and a 2024 survey and operator interview.

Domains	Attribute	Operational definition	Source
Performance	Simplicity	Percentage of surveyed sentinel staff rating the reporting process as simple or adequate	2024 sentinel hospital survey
Performance	No case notifications for >6 mo	Percentage of designated sentinel hospitals with no laboratory-confirmed bacterial gastroenteritis case notifications for >6 mo; distinct from missing scheduled weekly submissions	KDCA^a^ operational data
Performance	Acceptability	Distribution of responses on awareness of SSS operational goals	2024 sentinel hospital survey
Performance	Acceptability	Distribution of responses on overall satisfaction with SSS operation	2024 sentinel hospital survey
Performance	Timeliness	Delayed reports divided by all submitted reports; B/(A + B)	KDCA operational data
Reporting completeness	Submission completeness	All submitted reports divided by expected reports; (A + B)/C	KDCA operational data
Reporting completeness	On-time completeness	Reports submitted within 7 d divided by expected reports; A/C	KDCA operational data
Exploratory external comparison	Weekly temporal concordance	Pearson correlation between weekly all-pathogen SSS cases and NHI^b^ claim records (n=52 wk per y); not a sensitivity measure	KDCA and NHI claims
Exploratory external comparison	Age-stratified cross-category concordance	Pearson correlation between annual counts across 11 SSS surveillance targets or corresponding diagnosis code categories within each age group; not one-to-one pathogen validation	KDCA and NHI claims
Network allocation	Geographic allocation	Pearson correlation between population-proportional expected and actual sentinel site counts across 17 regions	KDCA and KOSIS^c^ population data

a KDCA: Korea Disease Control and Prevention Agency.

b NHI: National Health Insurance.

c KOSIS: Korean Statistical Information Service.

#### Simplicity

Simplicity captures the ease of structuring and operating the surveillance system [18]. We checked it in 3 aspects: case reporting, data collection, and dissemination of analytic results. Sentinel hospital personnel rated the simplicity of the case reporting process. The KDCA operator was asked about ease of data collection and result dissemination. Each item used a 3-point scale: simple, adequate, or complex.

#### Acceptability

Acceptability reflects how willing stakeholders are to take part in operations, reporting, and data use [18]. We measured it in 3 ways. The first was the proportion of sentinel hospitals with no laboratory-confirmed bacterial gastroenteritis case notifications for 6 months or longer in a year divided by the total number of sentinel hospitals multiplied by 100. This indicator concerned the absence of notified cases and was distinct from scheduled weekly report submission; a zero-case weekly submission counted toward completeness. The second was awareness of operational goals, asked as follows: “Are you aware of the operational goals of the surveillance system?” (responses: “yes,” “neutral,” or “no”). The third was overall satisfaction, asked as follows: “What is your overall satisfaction with the operation of the surveillance system?” (responses: “good,” “neutral,” or “poor”).

#### Timeliness

The KDCA expects sentinel hospitals to report cases within 7 days of diagnosis [13]. Broadly, timeliness refers to the time needed for reporting, analysis, and feedback within the SSS [18]. We measured it as the delayed reporting proportion: reports submitted after the 7-day window divided by the total number of reports submitted (the sum of on-time and delayed reports).

#### Completeness

If A denotes scheduled weekly submissions received within 7 days, B denotes delayed scheduled weekly submissions, C denotes the expected number of scheduled weekly submissions (number of designated sentinel hospitals multiplied by 52 weeks), and weekly submissions could report one or more cases or zero cases, we calculated submission completeness as (A + B)/C and on-time completeness as A/C. The delayed reporting proportion, our timeliness measure, was B/(A + B). These indicators were not arithmetic complements: timeliness was conditional on submissions that were received, whereas completeness used all expected submissions as the denominator. Reporting both forms of completeness separated failure to submit from delay among submitted weekly reports.

### Exploratory External Concordance

For exploratory external comparison, we aggregated SSS cases and NHI claim records across all 11 target categories by epidemiologic week and calculated a Pearson correlation using 52 paired weekly totals within each year. This ecological correlation asks whether the 2 data streams comove over weeks; it does not determine whether they identify the same patients or diagnoses. In a separate age-stratified analysis, we compared annual counts across 11 SSS surveillance target or corresponding diagnosis code categories within each age group (n=11 paired categories per panel). Because these correlations are sensitive to the highest-incidence categories, some parent *ICD-10* codes encompass more than one organism, and the claim definition is not laboratory confirmed, they were interpreted descriptively rather than as validation metrics.

### Geographic Allocation

For each of South Korea’s 17 administrative regions, the population-proportional expected number of sentinel hospitals was calculated as the total number of sentinel sites multiplied by the region’s share of the national resident population. Pearson correlation coefficients compared expected and actual site counts across regions for 2021 and 2023. This indicator assessed whether the network’s allocation followed population distribution; it did not evaluate regional disease incidence, regional reporting completeness, or geographic representativeness of reported cases.

### Statistical Analysis

Survey and operational data were summarized using frequencies, proportions, and means. Pearson correlation coefficients were calculated separately for weekly external concordance (52 paired weeks per year), age-stratified annual cross-category concordance (11 paired categories per age group and year), and geographic allocation (17 paired regions per year). Weekly coefficients were treated as descriptive because consecutive observations may be temporally autocorrelated and shared seasonality was not modeled. *P* values are reported descriptively and were not used to classify system performance; no adjustment for multiple exploratory correlations was applied. Analyses were conducted in SAS (version 9.4; SAS Institute).

### Reporting Guideline

We used the STROBE (Strengthening the Reporting of Observational Studies in Epidemiology) checklist for cross-sectional studies as a reporting aid for the survey and observational claim components [20]. A completed checklist is provided as Checklist 1. Because this study also included surveillance operational records and a single-operator interview, the STROBE checklist does not cover every component of the mixed methods evaluation.

### Ethical Considerations

The institutional review board of Kongju National University approved this study (approval number KNU_IRB_2024-072). Because we used deidentified, aggregated sentinel hospital data; an institutional interview with the KDCA operator; and anonymized NHI claim data, individual informed consent was waived. All data handling complied with the relevant Korean regulations on personal information and statistical data.

## Results

### Survey Results From Sentinel Hospitals

Of the 180 responding sentinel hospitals, 18 (10%) rated the reporting process as simple, 144 (80%) rated it as adequate, and 18 (10%) rated it as complex. Thus, 90% (162/180) rated reporting as simple or adequate (Table 3).

Responses on operational goal awareness and satisfaction were predominantly neutral. For awareness, of the 180 respondents, 55 (30.6%) answered “yes,” 97 (53.9%) were neutral, and 28 (15.6%) answered “no.” For overall satisfaction, 4.4% (n=8) rated the system as good, 79.4% (n=143) rated it as neutral, and 16.1% (n=29) rated it as poor (Table 3). These marginal distributions were analyzed separately; no within-respondent association among the 3 survey items was tested.

**Table 3.** Responses to the 2024 survey of staff responsible for bacterial gastroenteritis notification at 85.3% (180/211) of the sentinel hospitals in South Korea (n=180)^a^.

Items	Positive response, n (%)	Neutral or “adequate” response, n (%)	Negative response, n (%)
Rating of the reporting process	Simple: 18 (10)	Adequate: 144 (80)	Complex: 18 (10)
Awareness of the goals of the SSS^b^	Yes: 55 (30.6)	Neutral: 97 (53.9)	No: 28 (15.6)
Overall satisfaction with the SSS	Good: 8 (4.4)	Neutral: 143 (79.4)	Poor: 29 (16.1)

a Mean time taken to report cases per week 30.6 (SD 92.7) minutes.

b SSS: sentinel surveillance system.

### KDCA Operational Data

In 2023, a total of 2.8% (6/211) of the sentinel hospitals reported no laboratory-confirmed bacterial gastroenteritis cases for more than 6 months. This did not indicate missing scheduled weekly submissions; zero-case weekly submissions were counted in the completeness measures. The KDCA operator also reported that no formal standardized training program was provided for the role in 2023.

The delayed reporting proportion was 2.4% (255/10,652) of submitted weekly reports in 2021 and 1.1% (122/10,972) in 2023. Submission completeness was 99.9% (10,652/10,660) of expected weekly submissions in 2021 and 100% (10,972/10,972) in 2023. On-time completeness was 97.5% (10,397/10,660) and 98.9% (10,850/10,972) submissions in 2021 and 2023, respectively (Table 4).

**Table 4.** Operational reporting indicators, sentinel-reported cases, and National Health Insurance (NHI) claim record counts for 11 bacterial gastroenteritis diagnosis categories in South Korea (2021 and 2023)^a^.

Indicators	2021	2023
Sentinels in the surveillance system, n	205	211
Facilities with no laboratory-confirmed case notifications for >6 mo, n/N (%)	3/205 (1.5)	6/211 (2.8)
On-time scheduled weekly submissions received within 7 d (denoted as A), n	10,397	10,850
Delayed scheduled weekly submissions (denoted as B), n	255	122
Delayed reporting proportion (calculated as B/[A + B]), %	2.4	1.1
Expected scheduled weekly submissions (denoted as C [calculated as sentinel sites × 52 wk]), n	10,660	10,972
Submission completeness ([A + B]/C), %	99.9	100
On-time completeness (A/C), %	97.5	98.9
Total NHI claim records for the 11 bacterial gastroenteritis diagnosis categories, n	36,572	39,376
Health care facilities with NHI claim records for the 11 diagnosis categories, n	1030	1183
NHI claim records for patients aged 0‐6 y, n	8045	6307
Sentinel-reported cases for patients aged 0‐6 y, n	1333	1370

a Weekly submissions may include zero-case reports. The >6 month indicator denotes no laboratory-confirmed case notifications and is distinct from missing weekly submissions. NHI values are claim record counts and are not deduplicated by patient or episode; they must not be interpreted as incidence or directly compared with sentinel surveillance system case counts as if the counting units were equivalent.

### Exploratory External Concordance and Geographic Allocation

**Figure 1.**
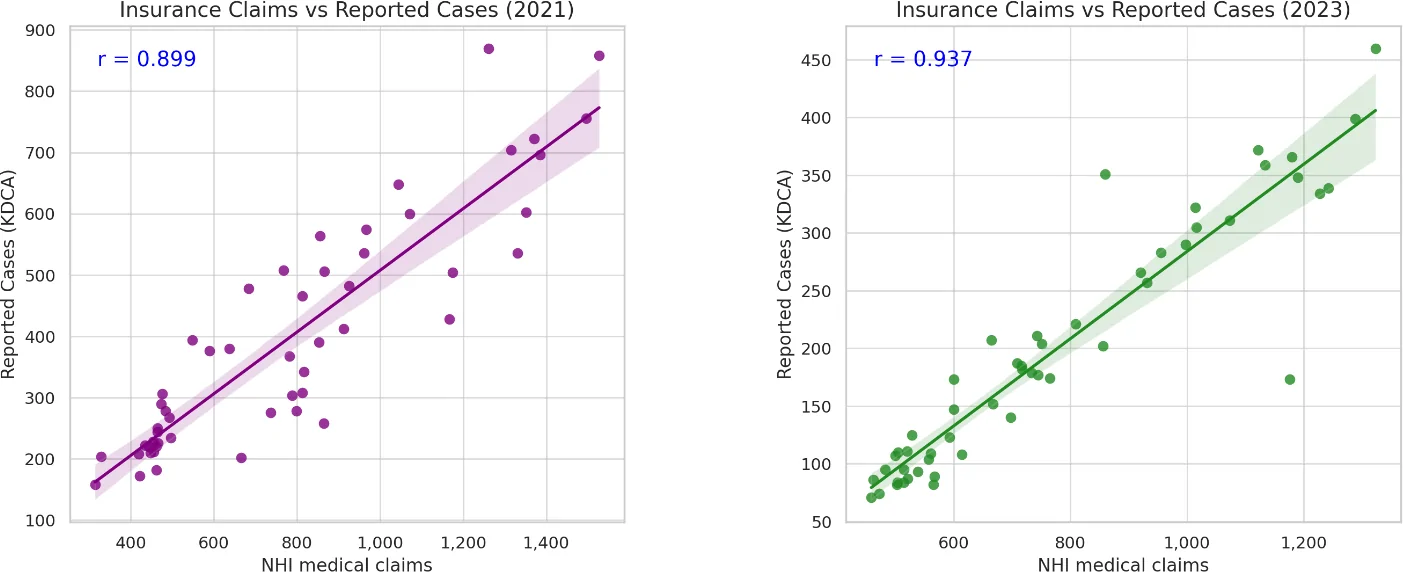
Weekly temporal concordance between total National Health Insurance (NHI) claim records carrying 1 of 11 bacterial gastroenteritis diagnosis codes and laboratory-confirmed cases reported through South Korea’s sentinel surveillance system shown separately for 2021 (A) and 2023 (B). Each point represents 1 epidemiologic week (n=52 per year); the line is the fitted linear relationship, and the shaded area is its 95% CI. The correlation is ecological and descriptive; shared seasonality and serial autocorrelation were not modeled, and the coefficient does not indicate diagnostic agreement or sensitivity. KDCA: Korea Disease Control and Prevention Agency.

Using 52 paired weekly all-pathogen totals per year, the exploratory Pearson correlation between SSS-reported cases and NHI claim records was 0.899 in 2021 and 0.937 in 2023 (Figure 1). These coefficients describe ecological weekly comovement and do not measure diagnostic agreement or surveillance sensitivity.

In the annual age-stratified cross-category analyses, each coefficient was based on 11 paired surveillance target or diagnosis code categories. The coefficients ranged from 0.665 to 0.969 in 2021 and from 0.939 to 0.983 in 2023. For children aged 0 to 6 years, the coefficients were 0.969 in 2021 and 0.982 in 2023; the largest 2023 coefficient was 0.983 in the group aged 7 to 12 years. The coefficients differed numerically between the 2 years, particularly in older age groups, but the nonconsecutive years and small number of category-level observations do not support a trend or causal interpretation. Full panels are shown in Multimedia Appendices 1 and 2.

The correlation between population-proportional expected and actual sentinel site counts across South Korea’s 17 administrative regions was 0.946 in 2021 and 0.951 in 2023 (*P*<.001 in both cases; Table 5), indicating close alignment of site allocation with population distribution.

**Table 5.** Summary of operational performance reporting completeness, exploratory external concordance, and geographic allocation for South Korea’s electronic bacterial gastroenteritis sentinel surveillance system (2021 and 2023)^a^.

Domains	Attribute	2021	2023	Interpretation
Performance	Reporting process rated as simple or adequate	—^b^	90% (162/180)	Predominantly simple or adequate
Performance	Awareness of operational goals; response distribution	—	Yes: 30.6% (55/180); neutral: 53.9% (97/180); no: 15.6% (28/180)	Predominantly neutral
Performance	Overall satisfaction; response distribution	—	Good: 4.4% (8/180); neutral: 79.4% (143/180); poor: 16.1% (29/180)	Predominantly neutral
Performance	Delayed reporting proportion	2.4% (255/10,652)	1.1% (122/10,972)	Low
Reporting completeness	Submission completeness	99.9% (10,652/10,660)	100% (10,972/10,972)	Near complete
Reporting completeness	On-time completeness	97.5% (10,397/10,660)	98.9% (10,850/10,972)	High
Exploratory external comparison	Weekly SSS^c^-NHI^d^ concordance (Pearson *r*; n=52 wk)	0.899	0.937	Strong descriptive ecological correlation
Exploratory external comparison	Age-stratified cross-category concordance (Pearson *r* for age of 0‐6 y; n=11 categories)	0.969	0.982	Strong exploratory correlation
Network allocation	Population-proportional vs actual sentinel site allocation (Pearson *r*; n=17 regions)	0.946	0.951	Strong alignment

a Neutral survey responses are reported separately and were not combined with positive responses. Correlation coefficients are descriptive. The sentinel surveillance system–National Health Insurance coefficients do not establish sensitivity, diagnostic agreement, one-to-one pathogen matching, or equivalence of counting units; the geographic coefficient assesses population-based site allocation only.

b No corresponding survey data were available for 2021; the survey was conducted in 2024 to evaluate the 2023 surveillance system.

c SSS: sentinel surveillance system.

d NHI: National Health Insurance.

## Discussion

### Principal Findings

Relative to the study objectives, the electronic SSS showed favorable operational performance. In 2023, a total of 90% (162/180) of respondents rated reporting as simple or adequate, 1.1% (122/10,972) of submitted weekly reports were delayed, submission completeness was 100% (10,972/10,972), and on-time completeness was 98.9% (10,850/10,972). Actual sentinel site allocation also closely followed population-proportional allocation across 17 regions. The exploratory external comparisons showed strong weekly ecological comovement between SSS cases and NHI claim records and generally strong annual cross-category correlations within age groups.

Acceptability findings were less favorable and more difficult to interpret. Only 30.6% (55/180) of respondents clearly reported awareness of the SSS’s operational goals, whereas 53.9% (97/180) selected “neutral” and 15.6% (28/180) selected “no.” Overall satisfaction was also mostly neutral (143/180, 79.4%), with 4.4% (8/180) rating it as good and 16.1% (29/180) rating it as poor. Because the awareness item did not state the operational goals, neutral or negative responses may reflect either limited awareness or ambiguity in the question. Nevertheless, clearer communication and routine onboarding are warranted. The absence of a standardized training program for the KDCA operator further supports the need for role-specific training and written operating procedures.

### Comparison With Prior Work

Systematic evaluation of surveillance systems can identify operational gaps and improve the information available to public health agencies. Although established frameworks exist, evaluations differ in the attributes assessed, data sources used, and analytic methods applied [19,21,22]. Our approach combined the US Centers for Disease Control and Prevention framework [19] with the KDCA-adapted framework [18], as in a previous evaluation of South Korea’s hand, foot, and mouth disease SSS [15]. Electronic notification systems have also been evaluated in other settings [23]. The present system likewise performed favorably for reporting simplicity, timeliness, and completeness while also revealing opportunities to improve stakeholder engagement and operator training.

Timeliness is a central surveillance attribute because delayed notification can reduce the usefulness of information for public health action [22]. NHI claims are not a substitute for real-time reporting because billing and data access processes introduce delay. The SSS, in contrast, requires notification within 7 days of diagnosis [13]. The 1.1% (122/10,972) delayed reporting proportion in 2023, therefore, indicates strong adherence to the specified reporting window. This metric does not measure onset-to-detection delay or outbreak detection speed, and its interpretation depends on the common 7-day threshold rather than pathogen-specific urgency [22,24]. Automated outbreak detection systems use different end points and require separate evaluation [25].

The external comparison requires more cautious interpretation. Surveillance sensitivity is the proportion of true community cases captured, which cannot be estimated without a credible denominator or reference standard. Other studies have compared gastroenteritis surveillance with web-based symptom reports or medication sales [26,27], and Korean claim data have been used to complement passive surveillance [28]. In our study, the weekly correlation used 52 ecological observations per year and indicates comovement of aggregate counts only. Consecutive weeks may be temporally autocorrelated, and shared seasonal patterns were not modeled; the coefficient should therefore be interpreted as descriptive comovement rather than an independent observation validation estimate. The age-stratified panels used 11 annual category-level observations and are particularly sensitive to leverage from high-incidence categories. Some *ICD-10* parent codes encompass more than one organism, further limiting pathogen-specific interpretation. Moreover, NHI claim records are not laboratory confirmed and were not deduplicated by patient or episode. Differences between the 2021 and 2023 coefficients, therefore, should not be interpreted as evidence that sensitivity changed. Validation of capture and diagnostic accuracy would require patient- or episode-level linkage to laboratory results or another microbiologically confirmed reference source. Geographic allocation should also be distinguished from case representativeness: our regional analysis assessed where sentinel hospitals were located relative to population, not whether reported disease incidence was representative.

The results support the operational value of a nationally coordinated electronic reporting platform, but they do not establish early outbreak detection performance, which was not evaluated. Potentially transferable design features include population-informed sentinel site allocation, standardized laboratory case definitions, and centralized electronic reporting. Site placement can influence outbreak detection and access to surveillance facilities [29,30]. Periodic comparison with administrative data may be useful as a quality assurance signal when interpreted as ecological consistency rather than as validation of cases or estimation of underreporting.

### Public Health and Informatics Implications

Electronic sentinel reports and administrative claims serve different functions. The SSS provides the timely, laboratory-based notifications required for routine surveillance. Claim data become available later and reflect reimbursement coding rather than confirmed infection. Their appropriate role in this context is retrospective quality assurance: marked divergence in weekly or category-specific patterns could prompt targeted review of coding, laboratory practice, or reporting workflows. Claim data alone cannot identify which source is correct, quantify underreporting, or replace laboratory-based surveillance.

Three practical improvements follow from the evaluation. First, routine feedback to sentinel hospitals could display reporting completeness and carefully labeled ecological concordance indicators, with alerts used to trigger review rather than grade performance automatically. Second, concise digital onboarding and standardized training materials should be provided to both sentinel personnel and KDCA operators. Third, future evaluations should obtain patient- or episode-level claims and link them, where legally and technically feasible, to laboratory-confirmed surveillance records. Such validation would directly address the case definition and duplicate use limitations identified in this study.

### Limitations

The principal limitation concerns the external comparator. NHI claims record diagnosis codes and billed procedures, not laboratory results, whereas SSS cases require laboratory confirmation. Claims may therefore include false-positive coding, empiric treatment, or unconfirmed testing. Accordingly, the correlations are interpreted only as exploratory ecological comparisons and not as a proxy for sensitivity.

Claims were counted at the record level and were not deduplicated by patient or clinical episode. Repeated use could alter weekly or category-specific patterns, particularly if revisit frequency varies by pathogen, age, or year. We did not compare absolute count levels, calculate incidence, or estimate capture fractions. Patient- or episode-level analysis is required before claims can be used for those purposes.

The survey response rate was 85.3% (180/211), but the characteristics of respondents were not formally compared with those of nonrespondents. Simplicity and acceptability estimates may therefore be affected by nonresponse and social desirability bias. We also did not test within-respondent associations among reporting simplicity, goal awareness, and satisfaction; the 3 marginal distributions should be interpreted separately. The operator interview involved 1 person, so it was used as contextual evidence rather than as a basis for generalizable quantitative inference.

The correlation analyses also have scope limitations. Weekly concordance was assessed in only 2 nonconsecutive years, so no continuous trend or pandemic effect can be inferred. Consecutive weekly observations may be temporally autocorrelated, and high coefficients may partly reflect shared seasonal patterns; seasonality and serial correlation were not modeled. Each age-stratified panel contained 11 category-level observations, several clustered near zero, and Pearson correlations were sensitive to high-incidence categories; multiple exploratory correlations were not adjusted for multiplicity. Some *ICD-10* parent codes encompass more than one organism, so the category-level comparisons do not imply one-to-one pathogen matching. The geographic analysis compared population-proportional and actual site allocation across 17 regions but did not compare regional disease incidence patterns.

Finally, our timeliness metric used a common 7-day threshold and did not measure the interval from symptom onset to detection or public health action. We did not evaluate outbreak detection algorithms, detection delay, flexibility, positive predictive value, stability, or cost-effectiveness [31,32]. These attributes require additional data and should be addressed in future evaluations.

### Conclusions

South Korea’s electronic SSS for bacterial gastroenteritis demonstrated favorable operational performance, including low reporting delay, near-complete submission, and population-aligned sentinel site allocation. Exploratory correlations with NHI claim records showed ecological comovement. Clearer communication of system goals, standardized operator training, and validation using deduplicated patient- or episode-level data linked to laboratory results are the most important next steps. These distinctions are relevant to other jurisdictions considering administrative data as a supplement to rather than a replacement for laboratory-based electronic surveillance.

## Supplementary material

10.2196/102373 Multimedia Appendix 1 Exploratory annual cross-category concordance between National Health Insurance claim records and sentinel-reported bacterial gastroenteritis cases within 6 age groups in South Korea in 2021.

10.2196/102373 Multimedia Appendix 2 Exploratory annual cross-category concordance between National Health Insurance claim records and sentinel-reported bacterial gastroenteritis cases within 6 age groups in South Korea in 2023.

10.2196/102373 Checklist 1 STROBE checklist.

## Abbreviations

ICD-10: International Classification of Diseases, 10th Revision
KDCA: Korea Disease Control and Prevention Agency
NHI: National Health Insurance
SSS: sentinel surveillance system
STROBE: Strengthening the Reporting of Observational Studies in Epidemiology
