# Supplementary material for: Performance of an Electronic Sentinel Surveillance System for Bacterial Gastroenteritis: Mixed Methods Evaluation Study
Source: JMIR Public Health Surveill. 2026 Aug 26;12:e102373. doi: 10.2196/102373 (PMC13513213; doi:10.2196/102373)
Supplement: Supplementary file 1 [file publichealth-v12-e102373-s001.docx]

Multimedia Appendix 1. Exploratory annual cross-category concordance between NHI claim records and sentinel-reported bacterial gastroenteritis cases within six age groups in South Korea, 2021.

Note. Each panel contains n=11 paired observations, one for each SSS surveillance target or corresponding diagnosis-code category. Some parent diagnosis codes may encompass more than one organism, several low-incidence categories cluster near the origin, and the coefficients are sensitive to the highest-incidence categories. Results are exploratory and should not be interpreted as sensitivity, diagnostic agreement, or one-to-one pathogen matching.
