# Supplementary material for: Performance of an Electronic Sentinel Surveillance System for Bacterial Gastroenteritis: Mixed Methods Evaluation Study
Source: JMIR Public Health Surveill. 2026 Aug 26;12:e102373. doi: 10.2196/102373 (PMC13513213; doi:10.2196/102373)
Supplement: Supplementary file 3 [file publichealth-v12-e102373-s003.docx]

**Adapted STROBE Checklist for Cross-Sectional and Observational Components**

*Performance of an Electronic Sentinel Surveillance System for Bacterial Gastroenteritis in South Korea: Mixed-Methods Evaluation Study*

**Note.** This checklist is used as a reporting aid for the cross-sectional survey and observational claims components. The study also includes surveillance operational records and a single-operator interview, which are not fully covered by STROBE. Page numbers refer to the revised manuscript with changes shown in blue.

| **Item** | **Section** | **Recommendation** | **Reported in manuscript** |
| --- | --- | --- | --- |
| **1a** | Title/Abstract | Identify the study design with a commonly used term in the title or abstract. | Title and Abstract, p 1. The title identifies a mixed-methods evaluation; the Abstract states the survey, operational-record, claims, and population components. |
| **1b** | Title/Abstract | Provide an informative, balanced summary of methods and findings. | Abstract, p 1. Data sources, years, denominators, main estimates, and limitations of claims inference are stated. |
| **2** | Background | Explain the scientific background and rationale. | Introduction, p 2. Global and Korean burden, surveillance context, and the evidence gap are described. |
| **3** | Objectives | State specific objectives and any prespecified hypotheses. | Introduction, p 2. Three objectives are stated; no causal hypothesis was prespecified. |
| **4** | Study design | Present key elements of the design early. | Methods—Study Design, p 2. Mixed-methods evaluation and separate operational/external-comparison components are described. |
| **5** | Setting | Describe setting, location, and relevant dates. | Methods—Data Sources, pp 2-3. South Korea, 211 sentinel hospitals, 2024 survey, and 2021/2023 records are specified. |
| **6a** | Participants | Give eligibility criteria and methods of selection. | Methods—Data Sources, p 2. All 211 designated sentinel hospitals were invited; staff responsible for notification were surveyed. |
| **6b** | Matched studies | For matched studies, describe matching criteria and numbers. | Not applicable. The study was not individually matched. Aggregate weeks, categories, and regions were paired only for ecological correlations. |
| **7** | Variables | Define outcomes, exposures, predictors, confounders, and effect modifiers. | Methods—Evaluation Framework, pp 4-5; Table 2, p 4. All operational indicators and external-comparison variables are defined. |
| **8** | Data sources/measurement | For each variable, give sources and assessment methods; describe comparability if more than one group. | Methods—Data Sources, Case Identification, and Evaluation Framework, pp 2-5. The different case definitions and counting units of SSS and NHI data are explicit. |
| **9** | Bias | Describe efforts to address potential sources of bias. | Methods—Case Identification, p 3; Limitations, pp 8-9. Primary/secondary codes with billed tests were used to improve coding specificity; residual misclassification, nonresponse, and non-deduplication are acknowledged. |
| **10** | Study size | Explain how study size was determined. | Methods—Data Sources, pp 2-3, and Statistical Analysis, p 5. Census invitation of 211 sites; available annual extracts determined 52 weeks, 11 categories, and 17 regions. |
| **11** | Quantitative variables | Explain how quantitative variables were handled and any groupings. | Methods—Evaluation Framework, pp 4-5. A/B/C denominators, six age groups, weekly totals, 11 categories, and 17 regions are defined. |
| **12a** | Statistical methods | Describe all statistical methods, including control of confounding. | Methods—Statistical Analysis, p 5. Frequencies, proportions, means, Pearson correlations, and descriptive P-value use are specified; no confounding model was used. |
| **12b** | Subgroups/interactions | Describe methods used for subgroups and interactions. | Methods—Exploratory External Concordance, pp 4-5. Age-stratified annual cross-category analyses are described. No interaction analysis was performed. |
| **12c** | Missing data | Explain how missing data were addressed. | Methods—Data Sources, p 2; Limitations, pp 8-9. 180/211 sites responded; no imputation or respondent/nonrespondent adjustment was performed. |
| **12d** | Sampling strategy | Account for sampling strategy when applicable. | Not applicable to the hospital survey because all designated sites were invited. Claims and operational extracts were nationwide/complete for the included years. |
| **12e** | Sensitivity analyses | Describe any sensitivity analyses. | No formal sensitivity analysis was performed. Limitations of the available record-level claims extract are stated in Methods and Limitations. |
| **13a** | Participants | Report numbers at each stage. | Results—Survey Results, p 5; Table 3, p 6. 211 invited and 180 respondents (85.3%). |
| **13b** | Nonparticipation | Give reasons for nonparticipation at each stage. | Reasons for survey nonresponse were not collected; this is acknowledged in Limitations, p 9. |
| **13c** | Flow diagram | Consider a flow diagram. | Not included because there was a single-stage census survey without participant exclusions after response. Counts are stated in text and Table 3. |
| **14a** | Descriptive data | Give participant characteristics and relevant variables. | Results, pp 5-6; Table 3, p 6. Survey response distributions and mean reporting time are reported. Facility characteristics were unavailable. |
| **14b** | Missing data per variable | Indicate numbers with missing data for each variable. | All 180 returned questionnaires contributed to the reported survey items; denominators are shown in Table 3. No item-level missingness was recorded in the analytic table. |
| **14c** | Follow-up time | Summarize follow-up time when relevant. | Not applicable; the survey was cross-sectional. Operational and claims analyses used complete calendar years. |
| **15** | Outcome data | Report numbers of outcome events or summary measures. | Results, pp 5-7; Tables 3-5, pp 6-7. Report counts, completeness, survey distributions, and correlation coefficients are presented. |
| **16a** | Main results | Give unadjusted and adjusted estimates with precision. | Results, pp 5-7; Tables 4-5 and Figure 1, pp 6-7. Analyses are descriptive and unadjusted. Pearson r and P values are reported where applicable. |
| **16b** | Category boundaries | Report category boundaries when continuous variables are categorized. | Methods—Exploratory External Concordance, pp 4-5. Age groups are listed as 0–6, 7–12, 13–18, 19–49, 50–64, and ≥65 years. |
| **16c** | Translate relative risk | Translate relative risk into absolute risk when relevant. | Not applicable. The study does not estimate relative risks or incidence. |
| **17** | Other analyses | Report subgroup, interaction, and sensitivity analyses. | Results, pp 5-7; Multimedia Appendices 1-2 (linked on p 10). Age-stratified exploratory analyses are reported; no interaction or formal sensitivity analysis was conducted. |
| **18** | Key results | Summarize key results with reference to objectives. | Discussion—Principal Findings, p 7. |
| **19** | Limitations | Discuss limitations, direction and magnitude of potential bias. | Limitations, pp 8-9. Claims misclassification, repeated records, nonresponse, single operator, ecological sample sizes, nonconsecutive years, and unassessed attributes are discussed. |
| **20** | Interpretation | Give a cautious interpretation considering objectives, limitations, multiplicity, and other evidence. | Discussion, pp 7-9. Operational conclusions are separated from exploratory claims correlations; causal and diagnostic interpretations are rejected. |
| **21** | Generalisability | Discuss external validity. | Conclusions, p 9. Transferable implications are limited to jurisdictions considering administrative data as a supplement to laboratory-based electronic surveillance. |
| **22** | Funding | State funding source and role of funder. | Funding, p 9. KDCA grant 2024-04-019 and the funder’s noninvolvement are stated. |
